# Genetic modifiers of Hb E/β^0 ^thalassemia identified by a two-stage genome-wide association study

**DOI:** 10.1186/1471-2350-11-51

**Published:** 2010-03-30

**Authors:** Richard Sherva, Orapan Sripichai, Kenneth Abel, Qianli Ma, Johanna Whitacre, Vach Angkachatchai, Wattanan Makarasara, Pranee Winichagoon, Saovaros Svasti, Suthat Fucharoen, Andreas Braun, Lindsay A Farrer

**Affiliations:** 1 Department of Medicine, Genetics Program, Boston University School of Medicine, (72 East Concord Street), Boston, (02118), USA; 2 Departments of Neurology, Genetics & Genomics, Epidemiology, and Biostatistics, Boston University Schools of Medicine and Public Health, (72 East Concord Street), Boston, (02118), USA; 3 Thalassemia Research Center, Institute of Molecular Biosciences, Mahidol University, (25/25 Phuttamonthon 4 Road), Salaya, Phuttamonthon, Nakornpathom, (73170), Thailand; 4 Department of Biochemistry, Faculty of Science, Mahidol University, (272 Rama VI Road, Ratchathewi District) Salaya, Phuttamonthon, Nakornpathom, (10400), Thailand; 5 Sequenom, Inc., (3595 John Hopkins Court), San Diego, (92121), USA; 6 Human Biomolecular Research Institute, San Diego, CA, USA; 7 Illumina, Inc., San Diego, CA, USA; 8 Dx Innovations, Inc. San Diego, CA, USA

## Abstract

**Background:**

Patients with Hb E/β^0 ^thalassemia display remarkable variability in disease severity. To identify genetic modifiers influencing disease severity, we conducted a two-stage genome scan in groups of 207 mild and 305 severe unrelated patients from Thailand with Hb E/β^0 ^thalassemia and normal α-globin genes.

**Methods:**

First, we estimated and compared the allele frequencies of approximately 110,000 gene-based single nucleotide polymorphisms (SNPs) in pooled DNAs from different severity groups. The 756 SNPs that showed reproducible allelic differences at *P *< 0.02 by pooling were selected for individual genotyping.

**Results:**

After adjustment for age, gender and geographic region, logistic regression models showed 50 SNPs significantly associated with disease severity (*P *< 0.05) after Bonferroni adjustment for multiple testing. Forty-one SNPs in a large LD block within the β-globin gene cluster had major alleles associated with severe disease. The most significant was bthal_bg200 (odds ratio (OR) = 5.56, *P *= 2.6 × 10^-13^). Seven SNPs in two distinct LD blocks within a region centromeric to the β-globin gene cluster that contains many olfactory receptor genes were also associated with disease severity; rs3886223 had the strongest association (OR = 3.03, *P *= 3.7 × 10^-11^). Several previously unreported SNPs were also significantly associated with disease severity.

**Conclusions:**

These results suggest that there may be an additional regulatory region centromeric to the β-globin gene cluster that affects disease severity by modulating fetal hemoglobin expression.

## Background

β-Thalassemia is a genetic disease in which an abnormal β-globin gene results in decreased (β+ thalassemia) or completely absent (β^0 ^thalassemia) production of the normal β-globin chain [1]. The hemoglobin E (HbE) allele, point mutation (G→A) in codon 26 (Glu→Lys) of the β-globin gene, can induce alternative splicing and thus result in decreased β-globin E chains [2,3]. Homozygosity for the HbE allele results in hypochromic microcytosis and minimal anemia, but the compound heterozygous condition, HbE/β^0 ^thalassemia causes a surprisingly variable anemia, ranging from nearly asymptomatic states to severe anemia and transfusion-dependency [4-6]. Hb E/β^0 ^thalassemia is most prevalent in and around Thailand, and with large numbers affected in other countries in Southeast Asian [7].

Several disease modifying hematological parameters have been identified, including fetal Hb (HbF),[8] red blood cell count (RBCC), Hb, mean corpuscular volume (MCV), mean corpuscular Hb (MCH), hemocrit, HbE,[9] and genetic factors contributing to the variability of some these parameters have been identified. HbF is the primary modifier of disease severity, because it can compensate for the lack of β-globin chains and HbA. There are now three known HbF QTLs, accounting for ~50% of the HbF heritability, *Xmn1*, *HBS1L-MYB*, and *BCL11A *[10-15]. There are other QTLs that appear to impact hematological parameters, including lymphotoxin alpha and tumor necrosis factor alpha associated with monocyte chemotactic protein-1 (MCP-1), [16] and a recent GWAS found SNPs on chromosomes 5 and 10 associated with MCV, SNPs on chromosomes 1, 4, 6, and 20 associated with RBCC, and numerous SNPs in the β-globin gene cluster and in erythrocyte membrane protein band 4.1-like 2 associated with hematocrit, HgB, MCH, MCV, and RBCC [17]. These QTLs for disease severity are biologically plausible but require confirmation.

Here, we attempt to identify variants that affect severity of HbE/β^0 ^thalassemia. An earlier study of SNPs in five genes known to influence globin gene expression and erythropoiesis failed to find significant associations with disease severity [18]. Subsequently, we observed strong association with 45 SNPs in a large linkage disequilibrium (LD) block containing the β-globin gene [19]. This association signal was ascribed to an effect of the *XmnI *polymorphism. To expand the search for severity modifying variants, we conducted a two-stage (pooled genome wide genotyping followed by individual genotyping in the top signals from stage one) association study on a sample of Thai Hb E/β^0 ^thalassemia patients.

## Methods

### Subjects

Sample collection, measurement of hematological data, and categorization of patients in this study were described elsewhere [18]. Briefly, a total of 1060 patients with Hb E/β^0^-thalassemia were ascertained from geographic regions across Thailand and a scoring system based on seven clinical criteria [18] was used to classify patients as mild (score = 0-3.5), moderate (score = 4-7) or severe (score = 7.5-10). Table 1 lists the clinical criteria and describes how they contribute to the severity score. Patients with a clinically intermediate form of the disease, detectable normal adult hemoglobin (HbA), other β- or α-globin mutation that known to affect disease severity such as (δβ)^0^-thalassemia or hereditary persistence of fetal hemoglobin, or α-thalassemia were excluded. Our analyses were based on the remaining patients who were classified as mild (n = 207) or severe (n = 305) disease. The study protocol was approved by the Institutional Review Board of Mahidol University and all subjects provided informed consent.

**Table 1:** Criteria and scoring system for classification of β^0^-thalassemia/Hb E patients

Criteria	Status	Score	Status	Score	Status	Score
Hb at steady state, g/dL	≥7.5	0	6.0--7.5	1	<6	2
Age at onset, years	>10	0	2--10	0.5	<2	1
Age at 1st transfusion, year	>10	0	4--10	1	<4	2
Transfusion requirement	Rare/none	0	Occasional	1	Regular	2
Spleen size, cm	<3	0	3--10	1	>10	2
Splenectomy	No	0	Yes	2		

### Hemoglobin Assays

Hemoglobin type and quantity were determined by automated hemoglobin cation exchange chromatography (Bio-Rad Variant, β-Thalassemia Short Program; Hercules, CA).

### Stage 1: Pooled Genotyping

Initially, two regionally matched pools were created, one representing participants with severe disease and another containing those with mild disease. For the severe disease pool, samples from individuals with the highest severity scores, calculated using the scoring system, were preferentially selected. The 395 unrelated samples were divided to create one DNA pool representing cases with mild symptoms (197 DNA samples) and one pool representing severe symptom (198 DNA samples). DNA was amplified with multiple displacement amplification (MDA) technology performed by Molecular Staging, Inc. (New Haven, CT). MDA allows for uniform genome amplification that results in minimal amplification bias across the genome (data not shown). To create equimolar pools of DNA, we used a stringent protocol for DNA concentration measurement using PicoGreen™ fluorescent dye in a Fluoroskan Ascent™ fluorometer.

The pooled genotyping was conducted by Sequenom, Inc. (San Diego, CA) using the MassARRAY platform. The MassARRAY platform for SNP scoring was complemented by a homogeneous, single-tube assay method (hME™ or homogeneous MassEXTEND™) in which two oligonucleotide primers anneal to and amplify a genomic target surrounding the polymorphism. A third primer (EXTEND), complementary to the amplified target up to but not including the polymorphism, was enzymatically extended one or a few bases through the polymorphic site by a DNA polymerase and then terminated by sequence-determined incorporation of dideoxynucleotides. PCR and MassEXTEND reactions for all assays were performed on each DNA pool. Organized in sets of 380 assays, two primer plates were combined to perform a PCR with each DNA pool. The PCR product plates were combined with the EXTEND primers, and the extension reaction was conducted. The obtained extension products were spotted four times onto a SpectroChip and measured with multiple rasters with a mass spectrometer. The measurements made on each chip were quantified using Sequenom's peak recognition and interpretation software. Areas under the peaks were calculated using optimized algorithms, and relative allele frequencies are estimated at 110,000 gene-based SNPs. The assayed SNPs had a median spacing of 10.4 kilobases and covered approximately 99% of all known and predicted human genes.

### Stage 2: Verification and Individual Genotyping

SNPs with allele frequencies suggesting significant differences (*P *< 0.02) across pools and were selected for verification by repeated pooled DNA analysis. This P-value threshold was chosen to limit the number of SNPs for individual genotyping and likewise the number of false positive association signals and also to provide the number of SNPs for individual genotyping specified in the study design. At this threshold, 808 SNPs showed reproducible allele frequency differences between disease severity pools and were individually genotyped using the MassARRAY™ system (Sequenom, Inc., San Diego, CA). For quality control purposes, 29 subjects were genotyped twice and one subject three times. Individuals and SNPs (N = 17) with substantial missing data (< 90%) were not considered for further analyses because these features usually indicate poor DNA quality and problems with genotype calls, respectively. Also, five X chromosomal SNPs with high heterozygosity in males (>1%) and nine SNPs showing inconsistencies in duplicated subjects or multiple Mendelian errors (>5%) were discarded for further analyses. Twenty-one other SNPs were also excluded because they had a low minor allele frequency, MAF, (<0.02 in both the case and control samples). Since all subjects in our study population have Hb E/β^0^-thalassemia and tests for Hardy-Weinberg equilibrium (HWE) in case-only populations are subject to many caveats [20], SNPs with significant deviation from HWE were not excluded. In total, 52 SNPs were dropped, leaving 756 for analysis.

### Statistical Analysis

The allele frequencies of the initial set of 110,000 SNPs were compared between pools containing individuals with mild disease vs. severe disease with Z tests with variances adjusted for variation in source DNA quantitation, diluting, and pipetting, as well as PCR/mass extension and chip dispensing/mass spectrometry measurement. The 756 individually genotyped SNPs which survived quality control were tested for association with the disease severity classification variable and also with HbF%, a quantitative measure known to affect disease severity. Associations with the binary disease severity outcome and HbF% were tested using logistic and linear regression models, respectively, adjusted for gender, age, and geographic region. Since HbF% measurements are not valid in regularly transfused patients, we only analyzed this trait in the mild disease group, who are not regularly transfused. The SNPs were modeled as the linear effect of the number of minor alleles. To correct for multiple testing, we employed a simple Bonferroni adjustment based on the 765 SNPs that survived quality control and were tested for association. We also computed a false discovery rate (FDR) for each *P*-value [21]. We present both the Bonferroni and FDR adjusted *P*-values to better illustrate the different levels of significance of the results. The FDR is an accepted measure of the significance of multiple association results, while we also state which findings pass the overly conservative Bonferroni threshold to highlight the highly significant findings. Finally, the linkage disequilibrium (LD) structure of the SNPs on chromosome 11, which included the β-globin gene cluster, was analyzed using Haploview [22].

## Results

The sample of 512 genotyped individuals was 51% female and represented five geographical regions in Thailand. Participants with mild disease were significantly older than those with severe disease (*P *= 0.0002), and males had significantly greater odds of severe disease than females (OR = 1.62, *P *= 0.01). The mean disease severity score was 2.1 among participants classified as having mild disease and 8.4 in those with severe disease. The ratio of HbE to HbF increases in regularly transfused patients, and likewise we observe a higher HbE to HbF ratio in the severe disease group who require more frequent transfusions. Table 2 shows the characteristics of the study sample by disease severity status, including demographic information and hematological measures. The mean age in the severe group was lower than the mild group, mostly because severe patients are identified at younger age. The difference in severity between males and females has no known biological explanation and likely represents random variation, an issue which we correct by adjustment. It should be noted that the hematological measures in the severe group are skewed from their genetically determined values because of the frequent transfusions in that group. Thus, no further analyses of these measures were performed in the severe group.

**Table 2:** Characteristics of the study sample by disease severity (N = 515)

Variable	Mild	Severe
Mean severity score (SD)	2.1 (1.0)	8.4 (0.8)
Mean Hb, g/dL (SD)	8.0 (1.4)	6.0 (1.1)
Mean HbF, % (SD)	40.7 (11.9)	31.1 (11.3)
Mean HbE, % (SD)	59.3 (11.9)	68.8 (11.3)
Mean HbE/HbF	1.8	2.7
Mean age, years (SD)	20 (14)	14 (9)
Female %	61	45
β-Thal Mutation, %		
Codon 17 (A>T)	23	25
Codon 41/42 (-TTCT)	40	47
IVS II-654 (C>T)	9	10
Hb Khon Kaen	9	1
IVS I-5 (G>C)	4	7
Codon 71/72 (+A)	0.50	4
Beta Deletion	4	1
Other	11	5

**Figure 1:**
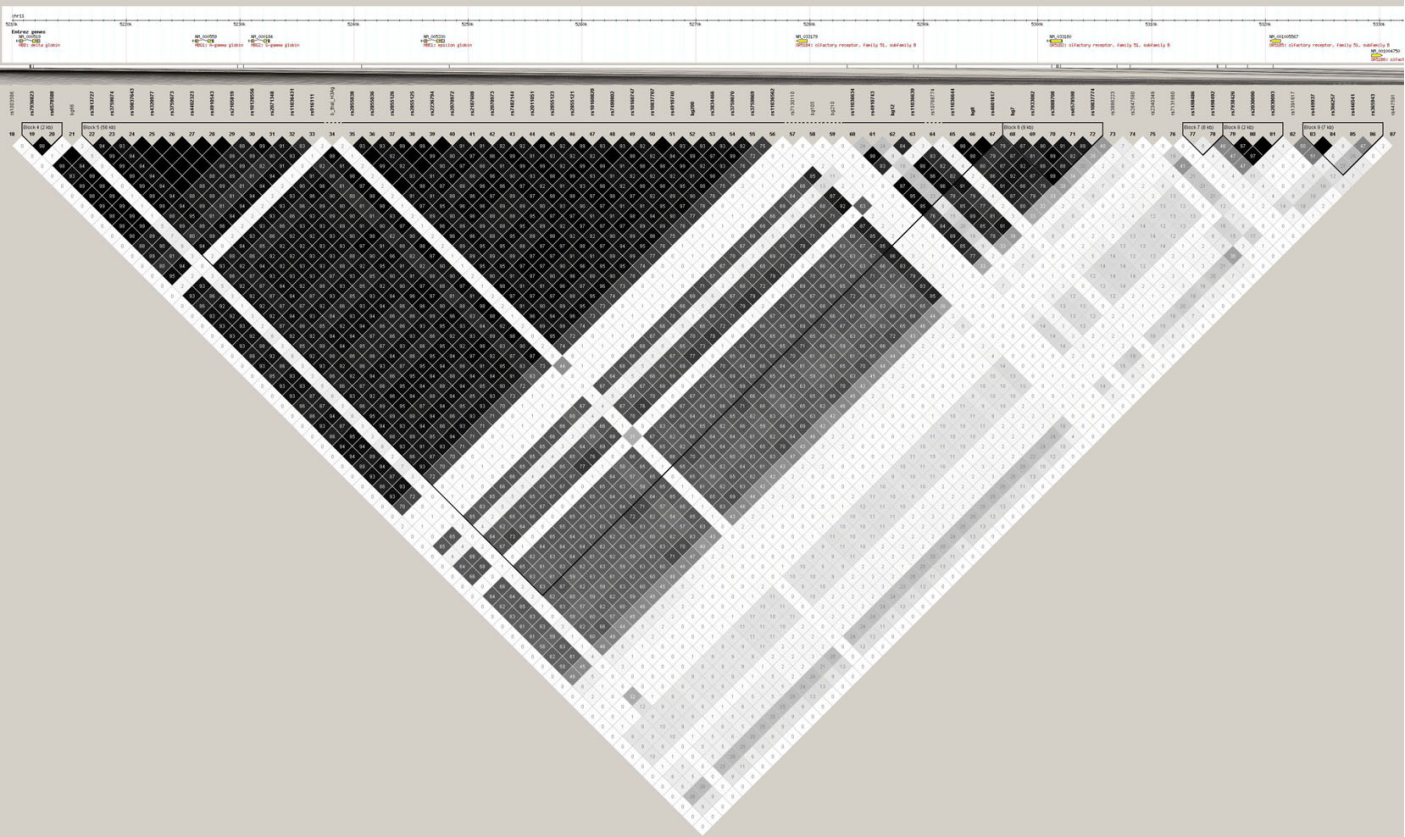
**Locations and linkage disequilibrium map structure of 70SNPs in the β-globin gene cluster and adjacent olfactory receptor genes**. The numbers in the squares represent the pairwise correlation (r^2^) between SNPs and darker fill indicates higher r^2^.

A total of 808 of the 110,000 SNPs showed reproducible allele frequency differences (*P *< 0.02) between the samples of pooled DNA from mild and severe cases, and these SNPs were genotyped in individual samples. Fifty-two of the genotyped SNPs were excluded for further analysis after quality control of the genotype data. Prior to correction for multiple testing, 377 of the 756 analyzed SNPs showed evidence for association with disease severity at *P *< 0.05, and 252 of these had a corresponding FDR = 5%. These included SNPs in the β-globin gene cluster on chromosome 11 previously reported to be associated with HbF levels in other samples, including the *Xmn1 *polymorphism 158 bp upstream from the Gγ gene and the *HincII *site in the ψβ-globin gene. Five of these SNPs were previously reported to be associated with disease severity in this sample by by Ma et al. [19] (see table 3). We observed an association with rs4376364 *HBS1L *(*P *= 4.3 × 10^-4^), although it was not significant after Bonferroni adjustment. Table 3 shows the allele frequencies, locations, and association results for the top 81 SNPs which had a FDR = 1%. Of these 81 SNPs, 50 remained significant after Bonferroni correction. LD analysis of the region on chromosome 11 encompassing 52 of the SNPs listed in table 3 revealed ten distinct haplotype blocks, the largest spanning 56.5 Kb and containing most of the significant SNPs (see Figure 1).

**Table 3:** SNPs associated disease severity at FDR less than 0.02

							Disease Severity					HbF%		
							
SNP	Chr	BP	Gene	Min	Maj	MAF	OR	*P*	Bonf	FDR	Beta	SE	*P*	Var
rs483850	1	203478366	FAIM3	T	A	0.45	0.65	9.7E-04	7.3E-01	0.01	0.05	.05	0.10	.52
rs934956	2	104555647	Intergenic	T	C	0.17	1.93	5.6E-04	4.2E-01	0.01	0.00	.05	0.97	.40
rs370898	3	21442705	ZNF659	G	T	0.05	0.37	1.4E-03	1	0.01	0.01	.05	0.93	.32
rs903047	3	132234707	NEK11	T	C	0.35	1.58	1.5E-03	1	0.01	-0.03	.05	0.44	.30
rs6443662	3	180834067	Intergenic	C	T	0.29	0.58	3.8E-04	2.8E-01	0.005	0.02	.05	0.57	.30
rs950310	4	9519119	SLC2A9	A	T	0.17	2.03	3.5E-04	2.6E-01	0.005	-0.08	.05	0.18	.29
rs258824	5	142577483	ARHGAP26	A	G	0.39	1.58	1.1E-03	8.3E-01	0.01	-0.06	.05	0.12	.29
rs234915	6	2154576	GMDS	A	C	0.21	0.52	1.3E-04	9.5E-02	0.002	0.03	.05	0.45	.27
rs545537	6	117751734	ROS1	A	G	0.17	1.90	8.4E-04	6.3E-01	0.01	0.03	.04	0.59	.27
rs4376364	6	135349178	HBS1L	G	T	0.34	1.70	4.3E-04	3.2E-01	0.01	-0.07	.05	0.10	.27
rs7804867	7	102627708	SLC26A5	A	G	0.28	1.76	3.4E-04	2.6E-01	0.005	-0.03	.05	0.51	.27
**rs6972505**	7	102657988	SLC26A5	C	G	0.24	2.13	7.6E-06	5.8E-03	0.0001	-0.04	.05	0.35	.27
rs1563408	7	113897140	FOXP2	G	A	0.49	1.63	4.1E-04	3.1E-01	0.01	0.02	.05	0.52	.27
**rs766003**	9	69709994	c9orf135	G	A	0.16	0.52	2.6E-04	1.9E-01	0.004	0.04	.05	0.37	.25
**rs7936823**	11	5206792	Intergenic	C	T	0.42	0.23	2.0E-11	1.5E-08	<.0001	0.10	.05	**0.04**	.25
**rs6578588**	11	5208867	Intergenic	A	G	0.42	0.21	1.2E-11	9.0E-09	<.0001	0.09	.05	0.07	.24
**rs3813727**	11	5212511	HBD	T	C	0.42	0.24	8.6E-11	6.5E-08	<.0001	0.10	.05	**0.04**	.23
**rs3759074**	11	5214371	Intergenic	T	C	0.41	0.18	3.6E-13 ±	2.7E-10	<.0001	0.10	.05	**0.05**	.23
**rs10837643**	11	5214635	Intergenic	A	T	0.43	0.22	5.4E-10	4.1E-07	<.0001	0.14	.05	**0.002**	.23
**rs4320977**	11	5214758	Intergenic	T	C	0.42	0.23	1.6E-11	1.2E-08	<.0001	0.10	.05	**0.04**	.22
**rs3759073**	11	5214862	Intergenic	A	G	0.42	0.22	2.5E-11	1.9E-08	<.0001	0.09	.05	0.07	.22
**rs4402323**	11	5215185	Intergenic	G	A	0.42	0.22	4.5E-12	3.4E-09	<.0001	0.10	.05	**0.04**	.22
**rs4910543**	11	5215423	Intergenic	C	G	0.42	0.22	5.8E-12	4.4E-09	<.0001	0.10	.05	**0.04**	.22
**rs2105819**	11	5216332	Intergenic	C	G	0.42	0.24	4.9E-11	3.7E-08	<.0001	0.11	.05	**0.03**	.22
**rs10128556 **	11	5220248	HBBP1	A	G	0.42	0.20	3.4E-13	2.6E-10	<.0001	0.10	.05	**0.05**	.21
***rs2071348***	11	5220725	HBBP1	C	A	0.41	0.19	4.8E-13	3.6E-10	<.0001	0.09	.05	0.07	.21
**rs11036431**	11	5222255	HBBP1	T	C	0.43	0.23	6.3E-12	4.8E-09	<.0001	0.08	.05	0.10	.20
**rs916111**	11	5225911	HBG1	A	T	0.43	0.23	1.7E-11	1.3E-08	<.0001	0.10	.05	**0.04**	.20
**rs2855038**	11	5228721	HBG1	A	G	0.43	0.24	4.5E-12	3.4E-09	<.0001	0.10	.05	**0.04**	.19
**rs2855036**	11	5229250	HBG2	A	G	0.41	0.20	3.5E-13	2.6E-10	<.0001	0.09	.05	0.08	.19
**rs2855126**	11	5229721	HBG2	G	C	0.43	0.24	7.0E-12	5.3E-09	<.0001	0.10	.05	**0.03**	.18
**rs2855125**	11	5230257	HBG2	A	C	0.43	0.25	1.2E-11	8.8E-09	<.0001	0.12	.04	**0.01**	.18
**rs2236794**	11	5230843	HBG2	G	A	0.42	0.24	2.5E-11	1.9E-08	<.0001	0.09	.05	0.07	.17
**rs2070972**	11	5231293	HBG2	T	G	0.43	0.24	3.6E-12	2.7E-09	<.0001	0.10	.03	**0.04**	.16
**rs2187608**	11	5231423	HBG2	G	C	0.41	0.20	7.0E-13	5.3E-10	<.0001	0.09	.04	0.06	.16
**rs2070973**	11	5231985	HBG2	A	G	0.44	0.23	5.6E-12	4.2E-09	<.0001	0.09	.05	0.06	.16
***rs7482144 *(XmnI)**	11	5232745	HBG2	T	C	0.42	0.20	3.7E-13	2.8E-10	<.0001	0.07	.05	0.17	.15
**rs2011051**	11	5233395	HBG2	C	A	0.43	0.24	1.2E-11	9.1E-09	<.0001	0.09	.05	0.07	.15
**rs2855123**	11	5233652	HBG2	T	A	0.44	0.24	4.9E-11	3.7E-08	<.0001	0.07	.05	0.14	.14
**rs2855121**	11	5233869	HBG2	A	G	0.41	0.20	2.2E-12	1.7E-09	<.0001	0.08	.05	0.12	.12
**rs10160820**	11	5239027	Intergenic	G	T	0.42	0.19	4.7E-13	3.6E-10	<.0001	0.09	.05	0.09	.12
**rs7480802**	11	5239080	Intergenic	A	G	0.43	0.24	2.1E-11	1.6E-08	<.0001	0.09	.05	0.07	.11
**rs10160747**	11	5240751	Intergenic	A	G	0.42	0.20	1.5E-12	1.2E-09	<.0001	0.09	.06	0.08	.11
***rs10837707***	11	5243234	HBE1	A	G	0.43	0.22	1.4E-12	1.0E-09	<.0001	0.09	.05	0.07	.11
**rs4910740**	11	5243705	HBE1	C	T	0.42	0.21	9.9E-13	7.5E-10	<.0001	0.10	.05	0.06	.11
bg200 *	11	5245549	HBE1	T	G	0.41	0.18	2.6E-13	2.0E-10	<.0001	0.10	.04	0.07	.10
**rs3834466**	11	5247976	HBE1	C	A	0.42	0.23	1.7E-11	1.2E-08	<.0001	0.07	.05	0.13	.10
**rs3759070**	11	5248038	HBE1	C	G	0.41	0.21	1.5E-12	1.1E-09	<.0001	0.08	.04	0.09	.08
**rs3759069**	11	5248240	HBE1	T	C	0.43	0.23	3.1E-12	2.4E-09	<.0001	0.09	.06	0.06	.08
***rs11036562***	11	5250549	HBE1	G	T	0.48	0.38	2.7E-08	2.0E-05	<.0001	0.07	.05	0.12	.08
**rs11036634**	11	5265154	Intergenic	A	G	0.50	2.34	1.4E-06	1.0E-03	<.0001	-0.02	.06	0.65	.07
bg12 *	11	5266933	Intergenic	G	T	0.49	2.31	6.8E-07	5.1E-04	<.0001	-0.06	.05	0.20	.06
***rs11036639***	11	5267001	Intergenic	C	T	0.46	0.34	6.0E-09	4.5E-06	<.0001	0.06	.04	0.21	.06
**rs11036641**	11	5267496	Intergenic	A	T	0.49	2.26	2.6E-06	2.0E-03	<.0001	-0.06	.04	0.21	.06
**rs11036644**	11	5268836	Intergenic	C	T	0.49	2.26	1.5E-06	1.2E-03	<.0001	-0.06	.05	0.18	.06
**rs4601817**	11	5268977	Intergenic	G	A	0.49	2.25	1.8E-06	1.4E-03	<.0001	-0.07	.05	0.17	.06
bg7 *	11	5270798	Intergenic	G	A	0.46	0.40	2.4E-07	1.8E-04	<.0001	0.05	.04	0.26	.06
**rs7933082**	11	5273186	Intergenic	C	G	0.48	0.47	1.5E-05	1.2E-02	0.0002	0.05	.04	0.36	.05
**rs3888708**	11	5274839	Intergenic	T	G	0.50	1.98	5.9E-05	4.5E-02	0.0009	-0.03	.04	0.47	.05
**rs6578598**	11	5275670	Intergenic	T	C	0.48	0.47	1.1E-05	8.4E-03	0.0002	0.04	.05	0.41	.05
**rs10837774**	11	5279964	OR51B4	A	G	0.45	0.38	7.3E-08	5.5E-05	<.0001	0.05	.04	0.32	.04
**rs3886223**	11	5300323	OR51B2	T	G	0.42	0.33	3.7E-11 ±	2.8E-08	<.0001	-0.01	.04	0.83	.04
rs2030090	11	5430669	OR51I2	G	A	0.22	1.81	1.5E-03	1	0.01	0.04	.05	0.46	.04
rs449937	11	5446921	OR51A10P	C	T	0.16	2.08	5.2E-04 ±	3.9E-01	0.01	-0.03	.05	0.64	.03
rs366257	11	5447119	OR51A10P	G	A	0.16	2.21	2.3E-04 ±	1.7E-01	0.003	-0.03	.04	0.58	.03
**rs446541**	11	5448876	OR51A10P	A	T	0.26	0.48	2.5E-05	1.9E-02	0.0004	0.002	.07	0.96	.03
rs678343	11	64728207	CAPN1	G	C	0.37	1.63	7.1E-04 ±	5.3E-01	0.01	0.04	.04	0.26	.03
rs593753	11	72369088	FCHSD2	T	C	0.29	0.60	6.5E-04 ±	4.9E-01	0.01	0.05	.04	0.21	.02
rs638135	11	85863336	ME3	A	G	0.23	0.59	1.1E-03 ±	8.3E-01	0.01	0.04	.06	0.32	.02
rs632538	11	85869341	ME3	G	A	0.25	0.60	1.5E-03 ±	1	0.01	0.04	.06	0.35	.02
rs2580874	12	8650077	AICDA	G	A	0.20	0.54	4.5E-04	3.4E-01	0.01	0.09	.05	0.04	.02
rs1045411	13	29931232	HMGB1	A	G	0.29	0.58	3.9E-04	2.9E-01	0.005	0.02	.05	0.70	.01
rs886599	14	70244197	Intergenic	A	G	0.10	2.70	1.2E-04	9.0E-02	0.002	0.04	.08	0.64	.01
rs1160027	17	51173101	Intergenic	T	G	0.20	0.56	8.5E-04	6.4E-01	0.01	-0.01	.06	0.79	.01
rs953695	18	59541550	SERPINB11	T	G	0.21	1.83	7.6E-04	5.8E-01	0.01	-0.03	.04	0.63	.01
rs243341	19	4356106	CHAF1A	T	C	0.41	1.59	1.5E-03	1	0.01	-0.04	.04	0.33	.00
rs379327	19	61061978	NLRP4	A	G	0.15	1.97	7.8E-04	5.9E-01	0.01	-0.07	.04	0.26	.00
rs639763	20	33325318	MMP24	T	C	0.30	0.57	3.9E-04	2.9E-01	0.005	-0.04	.07	0.35	.00
rs6625978	X	71201690	PIN4	C	A	0.06	3.35	1.3E-03	1	0.01	-0.001	.04	1.00	.00

BP = location of SNP in base pairs in NCBI build 36

Min = Minor allele

Maj = Major allele

Beta = Absolute change in HbF% on the log scale per copy of the minor allele

SE = Standard error of the Beta estimate

Bonf = Bonferroni adjusted *P *--value

FDR = false discovery rate

MAF = minor allele frequency

* = SNP not in NCBI database

± = Chromosome 11 SNPs associated with disease severity at P < 0.05 after adjustment for Xmn1

SNPs significantly associated with disease severity after Bonferroni correction are in bold

*P*-values for SNPs associated with HbF% at *P *≤ 0.05 in the mild disease group are in bold

SNPs in italics were previously shown to be associated with disease severity in this sample by Ma et al. 2007 [19]

Var = Percent of the variation in log transformed HbF% explained by each SNP

In addition to the previously reported associations with SNPs in the β-globin cluster, we identified association signals with seven SNPs in the region upstream (centromeric) from the β-globin cluster that were not in the same haplotype block. Rs3886223 was the most significantly associated SNP in this group, with the common allele contributing to increased risk of severe disease in an additive fashion (OR = 3.03, *P *= 2.82 × 10^-8^). The association with this SNP remained significant after adjustment for SNPs in the β-globin cluster, including *Xmn1*, suggesting that the biological variant tagged by this SNP is distinct from the one in the β-globin cluster. *Xmn1 *is correlated with rs3759074 at r^2 ^= .92 and with rs3886223 at r^2 ^= .46. This region contains many olfactory receptor genes and rs3886223 is less than 500 bp upstream from *OR51B2*.

Only one result among SNPs on other chromosomes remained significant after Bonferroni adjustment; rs6972505 (OR = 2.13, *P *= 7.6 × 10^-6^) is in an intron of solute carrier family 26 member 5 (*SLC26A5*) located on chromosome 7. A second SNP, rs234915 in the GDP-mannose 4,6-dehydratase gene (*GMDS*) on chromosome 6, was not significant after Bonferroni adjustment but had a significant FDR (OR = 1.92, *P *= 1.3 × 10^-4^), with the major allele associated with severe disease. Several other SNPs had a FDR less than 0.01 (see Table 3). Tests for genetic association with HbF% within the mild disease group revealed nominally significant results with 14 SNPs in the β-globin gene cluster (Table 3).

## Discussion

A previous study of a Hong Kong Chinese population showed HbF to be 80-90% heritable, [12] and previous studies have shown that *Xmn1 *accounts for approximately 13% of the variation in HbF in normal Europeans [11] and for a similar proportion in a Chinese sample heterozygous for the β-thalassemia mutation [12]. In addition, about 15% of the HbF variance was explained by a polymorphism in *BCL11A *on 2p15, 19% by an intergenic variant in *HBS1L-MYB *on 6q23, and 10% by *Xmn1 *in Europeans without hemoglobinopathies [10]. Assuming a maximum of 50% of the total trait variance has been explained, a substantial number of QTL contributing to the heritability of HbF remain undiscovered. By studying a disease severity phenotype we have the opportunity to identify variants that affect disease through HbF as well as other pathways. In this study, we replicated association findings from previous studies of several disease-modifying SNPs in a Thai population with Hb E/β^0 ^thalassemia and report associations with SNPs that may represent additional β-globin gene regulatory regions or that alter disease severity through novel pathways.

This study is the first whole genome association scan for modifiers of Hb E/β^0 ^thalassemia. The two-stage study design allowed for cost effective identification of a targeted set of SNPs for individual genotyping. The fact that most of the SNPs identified as having significant differences in allele frequency between severity groups in the analysis of pooled DNA samples also showed nominally significant associations with severity after individual genotyping demonstrates the utility of a two-stage design.

Our study has several limitations. Since the pooled genotyping was done on a marker panel that is less dense than ones used in many contemporary genome wide association studies, we do not have the same level of genome coverage provided by other genotyping platforms. While our use of a gene-based SNP panel likely reduced the number of false negative results due to low marker density, the fact that no SNPs were typed in the *BCL11A *region, a well-confirmed modifier of HbF level, [11-15] underscores the gain in information afforded by the high density SNP arrays. In any case, the low marker density does not negate the highly significant associations we did identify, particularly those independent of the β-globin gene region. The SNPs allelotyped in the discovery phase are the set of marker assays developed at Sequenom and demonstrated to be polymorphic in Caucasians. Thus, prior to this study, the MAFs of these SNPs were unknown in the Thai population. Similar to all GWA studies of non-Caucasians using SNP chips developed based on marker allele frequencies in Caucasians, some SNPs were not polymorphic and hence a true association in this circumstance would not be detected.

The observation that several SNPs within the β-globin gene cluster were more significantly associated than Xmn1 with disease severity does not imply that one of these variants rather than XmnI has a causal effect on disease severity. There is a substantial body of literature suggesting Xmn1 has a functional role in determining HbF% and F-cell number [11,12,19] and there is no such evidence for any of these other SNPs Alternately, this observations may be due to *Xmn1 *allele frequency differences across populations. For example, the frequency of the *Xmn1 *minor allele is much higher in this sample (42%) than in most other populations studied, including a sample of unaffected Hong Kong Chinese parents of offspring with β-thalassemia (17%),[12] an unselected group of 720 UK twins (33%),[10] the African American Cooperative Study of Sickle Cell Disease (7%),[23] a Brazilian sickle cell disease study sample (0%),[23] and a group of 58 Chinese β-thalassemia patients (5%) [24]. Although there are small differences in significance among SNPs across the β-globin gene cluster, all of these SNPs have similar minor allele frequencies and are highly correlated, and it is not possible to identify definitively all causal variants in the β-globin cluster by genetic association analysis. Functional studies are needed to delineate the spectrum of independent variants in the β-globin region with a causal effect on disease severity and HbF level.

The associations observed with SNPs in the olfactory receptor cluster, which is adjacent to but distinct from the β-globin cluster, are novel and may represent a new disease modifying region. The biological connection between olfactory receptors, which interact with odorant molecules in the nose to initiate a neuronal response that triggers the perception of smell, and severity of Hb E/β^0 ^thalassemia is not obvious. However, these SNPs (nearly all of which are located in regulatory regions), or variants strongly associated with them, may be acting as cis-regulatory elements [25] and thus down-regulate transcription of HbF or impact disease severity by altering expression of other β-globin genes. This hypothesis is consistent with the observation that at least one of these genes is expressed in human erythroid cells at all stages of development [26]. High-level transcription of the globin genes through enhancement by a remote cis element, the locus control region (LCR), may involve chromatin remodeling [27] including the region containing the olfactory receptor genes. As these findings are novel, they have not been replicated in an independent sample. Replication of these associations for either disease severity or HbF is necessary.

The findings with SNPs in SLC26A5 and GDMS are also novel and need to be replicated in independent samples. SLC26A5 acts as the primary motor molecule in cochlear outer hair cells due to its ability to undergo voltage-induced conformational change [28]. Although no pathway through which this gene might affect thalassemia severity is immediately apparent, the *SLC26A5 *gene footprint partially overlaps the proteasome 26S subumit gene (*PSMC2*), which immunoprecipitates with transcription factors for RNA polymerase II in rats, indicating a role in transcriptional regulation [29]. GDMS has functions related to embryonic development and in regulation of the immune response [30]. Finally, of note there was only one individually typed SNP in the region containing *BCL11A*, and it was 30 Kb from the gene. The SNP was removed due to low call rate, but it was nominally associated with HbF% in the mild group (*P *= 0.01).

## Conclusions

These results suggest that there may be an additional regulatory region centromeric to the β-globin gene cluster that affects disease severity by modulating fetal hemoglobin expression. Also, the replication of previous findings related to HbF highlight the utility of pooled genotyping as a cost effective first step to identify a subset of SNPs for individual genotyping.

## Competing interests

The authors declare that they have no competing interests.

## Authors' contributions

*Study concept and design: *KA, PW, SF, AB, LF. *Acquisition of data: *KA, OS, JW, VA, WM, SF, AB. *Analysis and interpretation of data: *RS, QM, LF. *Drafting of the manuscript: *RS, QM, LF. *Critical revision of the manuscript for important intellectual content: *RS, PW, SF, AB, LF. *Statistical expertise*: RS, QM, LF. *Obtained funding: *SF, AB, LF. *Administrative, technical, or material support: *OS, JW, VA, WM, PW, SS, SF, AB, LF. *Study supervision *SF, AB, LF. All authors read and approved the final manuscript.

## Pre-publication history

The pre-publication history for this paper can be accessed here:

http://www.biomedcentral.com/1471-2350/11/51/prepub
